# NiCu Alloy Nanoparticle-Loaded Carbon Nanofibers for Phenolic Biosensor Applications

**DOI:** 10.3390/s151129419

**Published:** 2015-11-20

**Authors:** Dawei Li, Pengfei Lv, Jiadeng Zhu, Yao Lu, Chen Chen, Xiangwu Zhang, Qufu Wei

**Affiliations:** 1 Key Laboratory of Eco-Textiles, Ministry of Education, Jiangnan University, Wuxi 214122, China; E-Mails: ldw19900323@163.com (D.L.); vl1403979894@sina.com (P.L.); 2 Fiber and Polymer Science Program, Department of Textile Engineering, Chemistry and Science, North Carolina State University, Raleigh, NC 27695-8301, USA; E-Mails: jzhu14@ncsu.edu (J.Z.); ylu14@ncsu.edu (Y.L.); cchen20@ncsu.edu (C.C.)

**Keywords:** carbon nanofibers, NiCu alloy nanoparticles, electrospinning, laccase, phenolic biosensor

## Abstract

NiCu alloy nanoparticle-loaded carbon nanofibers (NiCuCNFs) were fabricated by a combination of electrospinning and carbonization methods. A series of characterizations, including SEM, TEM and XRD, were employed to study the NiCuCNFs. The as-prepared NiCuCNFs were then mixed with laccase (Lac) and Nafion to form a novel biosensor. NiCuCNFs successfully achieved the direct electron transfer of Lac. Cyclic voltammetry and linear sweep voltammetry were used to study the electrochemical properties of the biosensor. The finally prepared biosensor showed favorable electrocatalytic effects toward hydroquinone. The detection limit was 90 nM (S/N = 3), the sensitivity was 1.5 µA µM^−1^, the detection linear range was 4 × 10^−7^–2.37 × 10^−6^ M. In addition, this biosensor exhibited satisfactory repeatability, reproducibility, anti-interference properties and stability. Besides, the sensor achieved the detection of hydroquinone in lake water.

## 1. Introduction

Nowadays, metal nanoparticles have become widely utilized in electrochemical sensing and biosensing applications, which can be attributed to their rich electronic properties, high surface area, great mechanical properties, and excellent chemical stability [1]. Among various metals, nickel (Ni) nanoparticles and copper (Cu) nanoparticles are inexpensive and widely available. Many researchers have reported nickel nanoparticle [2,3,4,5] and copper nanoparticle [6,7,8,9] biosensors. Although Ni and Cu nanoparticles have shown significant electrocatalytic ability toward some substrates, e.g., glucose, pure Ni and Cu nanoparticles are not stable under highly oxidizing conditions [10], while NiCu bimetallic alloy nanoparticles can overcome this disadvantage, leading to higher stability as compared to monometallic nanoparticles. In addition, due to the synergistic bimetallic electrocatalysis, NiCu alloy nanoparticles have also shown higher electrochemical responses than pure Ni or Cu [11]. Hence, the use of NiCu bimetallic alloy nanoparticles in biosensors it is favorable.

Various techniques have been employed to prepare alloy nanoparticles, including electrodeposition [12], laser ablation deposition [13], hydrogen reduction [14], and chemical vapor deposition [15]. Electrospinning, as a facile process technique for the production of nanofibers, can also be used to prepare alloy nanoparticles by carbonizing electrospun metal precursor-containing polyacrylonitrile (PAN) nanofibers [16,17]. In this approach, metallic alloy nanoparticles can be uniformly dispersed in the carbon nanofibers (CNFs), which is favorable for the electrocatalytic performance of metallic nanoparticles [18]. In addition, electrospun CNFs possess high conductivity, large surface porosity and good biocompatibility, and have easily functionalized surfaces [19]. CNFs have been investigated for use in biosensors, and studies revealed that the CNFs could play a role of “molecule wire” connecting electrode surfaces and the active centers of biological molecules during the direct electron transfer process of the biosensors [20,21,22,23]. For example, Guo *et al*. prepared Pd-Ni/CNF composites and found that Pd-Ni alloy showed improved electrocatalytic activity for sugar oxidation [18].

Hydroquinone is a type of environmental pollutant that can be found in the human diet, medicines and cosmetics [24]. It is very hazardous to animals, plants and human beings when it exists in the environment, even at very low concentration [25]. As a result, the search for highly efficient detection methods for hydroquinone has attracted more attention recently. Laccase (Lac) can oxidize phenolic compounds, and based on this, many Lac-based biosensors for the detection of phenols have been reported [26,27,28,29]. Compared with conventional detection methods, Lac- or enzyme-based biosensors display more advantages, such as facile, simple, fast techniques and amenability to miniaturization which can enable *in situ* detection.

In this study, we prepared NiCu alloy nanoparticle-loaded carbon nanofibers (NiCuCNFs) by a combined electrospinning and thermal treatment method. Then, a NiCuCNFs, Lac and Nafion mixture was deposited on a glass carbon electrode (GCE) surface to construct a novel biosensing platform. The NiCuCNFs helped achieve the direct electron transfer (DET) of Lac. Meanwhile, the obtained biosensor was used to detect hydroquinone and showed low detection limit, high sensitivity, good selectivity, and excellent stability. Moreover, the biosensor demonstrated that it can be applied to the detection of hydroquinone in real water environments.

## 2. Experimental Section

### 2.1. Chemicals and Reagents

Laccase was purchased from Sigma-Aldrich (St Louis, MO, USA). Polyacrylonitrile (PAN, M_w_ = 150,000 g·mol^−1^) was obtained from Pfaltz & Bauer Inc. (Waterbury, CT, USA). *N*,*N*-dimethylformamide (DMF), hydroquinone, Nafion (10 wt % dispersion in water), copper acetate and nickel nitrate were also all obtained from Sigma-Aldrich (St Louis, MO, USA). PH 5.0 0.1 M acetate buffer solution was prepared as the test solution.

### 2.2. Synthesis of NiCuCNFs

NiCuCNFs were prepared by the processes of electrospinning and carbonization. Firstly, an electrospinning solution was prepared by mixing 10 wt % of PAN, 2 wt % copper acetate and 2.9 wt % nickel nitrate in 20 mL DMF with a copper acetate/nickel nitrate molar ratio of 1/1 under stirring for 12 h. Then, the obtained mixture solution was transferred to a syringe and electrospun into the precursor nanofibers. The electrospinning parameters were as follows: work distance 20 cm, voltage 20 kV, flow rate 1 mL·h^−1^. Finally, electrospun precursor nanofibers were put in a muffle furnace to conduct the carbonization process to prepare NiCuCNFs. The pre-oxidation temperature was 250 °C, stabilization time was 2 h, heating rate was 5 °C·min^−1^, the whole pre-oxidation process was maintained in an air atmosphere. The carbonization temperature was 900 °C, carbonization time was 2 h, heating rate was 2 °C·min^−1^, the whole process was kept under a nitrogen atmosphere. For comparison, NiCNFs and CuCNFs were also prepared while keeping the metal salt content constant.

### 2.3. Preparation of Biosensors

Lac (3 mg·mL^−1^), Nafion (1.5 wt %) and NiCuCNFs (0.3 mg·mL^−1^) were mixed together and coated onto a polished GCE surface to fabricated the biosensor. During the process, NiCuCNFs were firstly dispersed into acetate buffer with the help of ultrasonication. NiCuCNFs suspension, 40 µL of Nafion and 15 mg of Lac was then mixed and magnetically stirred for 1 h. Eventually, 10 µL of the mixture was coated onto the polished GCE surface to fabricate a Lac-NiCuCNF-Nafion modified GCE (Lac-NiCuCNF-Nafion/GCE). The dried Lac-NiCuCNF-Nafion/GCE was stored at 4 °C.

For comparison, Lac-Nafion/GCE, NiCuCNF-Nafion/GCE, Lac-CuCNF-Nafion/GCE and Lac-NiCNF-Nafion/GCEs were also prepared while keeping Nafion, Lac and nanofiber content constant. Before electrochemical measurements, all the modified electrodes were immersed into buffer solution for 0.5 h to remove unstable substances.

### 2.4. Structure Characterization

Field emission scanning electron microscopy (FESEM, FEI Quanta 3D, Hillsboro, OR, USA) and a high-resolution transmission electron microscope (HR-TEM, FEI Titan 80-300, Hillsboro, OR, USA) were employed to observe the morphology of NiCuCNFs. A SuperX Energy Dispersive Spectrometry (SuperX EDS, Hillsboro, OR, USA) was used to characterize the elemental mapping of NiCuCNFs. X-ray diffraction (XRD, Rigaku Smartlab, Tokyo, Japan, Cu Kα, λ = 1.544 Å) in a 2θ range of 10°–80°) was employed to analyze the chemical compositions of NiCuCNFs.

### 2.5. Performance Evaluation

Electrochemical experiments were conducted using a DY2300 potentiostat (Digi-Ivy, Inc., Austin, TX, USA). The electrochemical tests were carried out by using a three-electrode cell, the biosensor electrode was used as the work electrode, platinum wire was used as auxiliary electrode and Ag/AgCl electrode was used as reference electrode. For the linear sweep voltammetry experiments, the experiment parameter was set as following: linear frequency 60 Hz, scan rate 0.1 V·s^−1^, and sensitivity 1 × 10^−5^ A·V^−1^. The buffer solution was deoxidized for 20 min before all of electrochemical experiments to create a nitrogen ambient except the linear sweep voltammetry experiments for the biosensor.

## 3. Results and Discussion

### 3.1. Characterization

**Figure 1:**
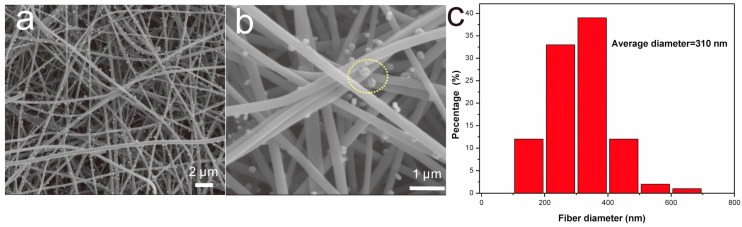
(**a**,**b**) SEM image of NiCuCNFs and (**c**) fiber diameter distribution of NiCuCNFs.

Figure 1 shows SEM images of NiCuCNFs. As shown in Figure 1a, the NiCuCNFs formed a porous net-like structure with random distribution. Meanwhile, numerous NiCu alloy nanoparticles could be observed on the surface of NiCuCNFs. It is seen from Figure 1b that the NiCu alloy nanoparticles were attached on the surface of nanofibers. Specially, as the part marked by yellow circle, the NiCu alloy nanoparticles exhibited a hexagonal structure. The average fiber diameter of NiCuCNFs was *ca.* 310 nm (Figure 1c).

**Figure 2:**
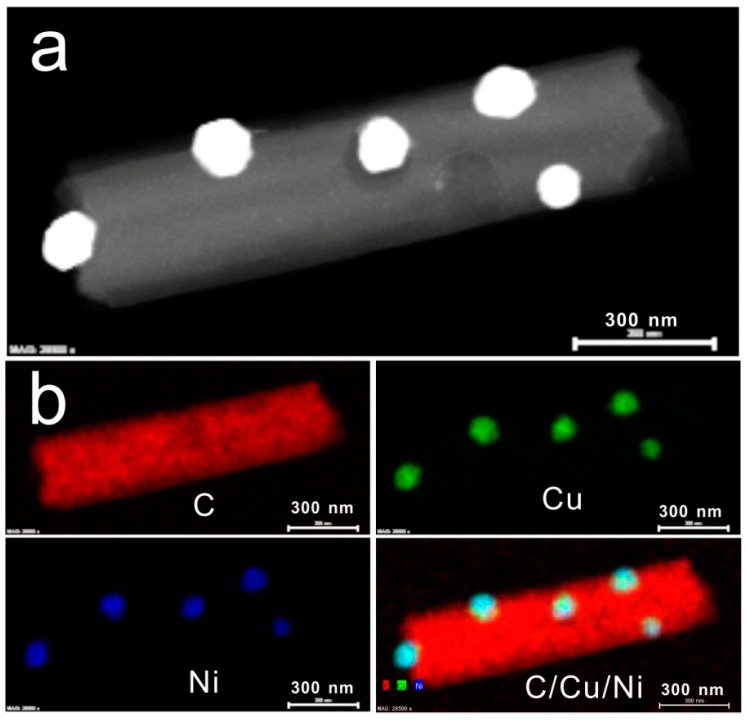
(**a**) Typical TEM image and (**b**) EDS elemental maps of NiCuCNFs.

Figure 2 shows the typical TEM image of NiCuCNFs and the corresponding elemental mapping. It can be seen from Figure 2a that the nanoparticles were all attached on the outside surface of NiCuCNFs with uniform distribution. Based on this specific structure, the NiCu alloy nanoparticles can have easy access to catalyze substances. The average diameter of the nanoparticles was about 60 nm. Figure 2b shows the EDS elemental maps of NiCuCNFs. It confirms that the NiCuCNFs was consisted of C, Cu and Ni elements and NiCu nanoparticles were dispersed on the surface of CNFs.

**Figure 3:**
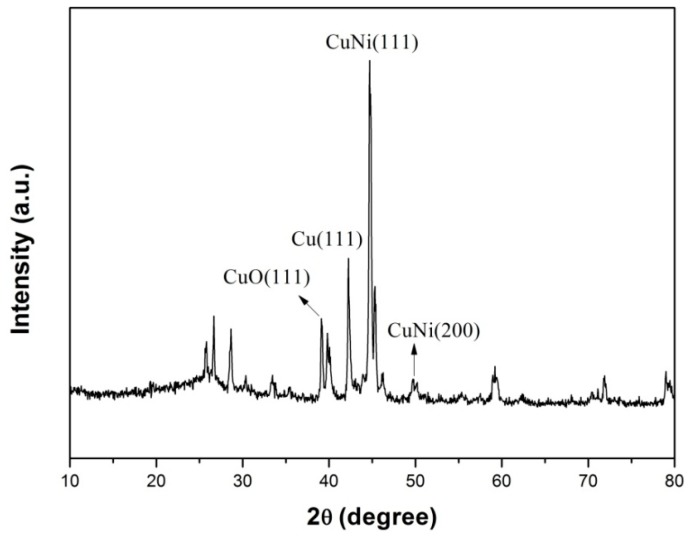
XRD pattern of NiCuCNFs.

XRD characterization was used to characterize the chemical constitution of NiCuCNF. Figure 3 displays the XRD pattern of the NiCuCNFs. As shown in Figure 3, numerous peaks can be observed and among these peaks, the peak appearing at *ca.* 25.6° was ascribed to the (002) plane of carbon [30]. The peaks at *ca.* 44.1° and 50.9° were respectively assigned to the crystal planes (111) and (200) of NiCu alloy, which was identical with the reported result [31]. Besides, the peak at about 43.2° was ascribed to the (111) crystal plane of Cu, and the peak at around 38.6° can be related to the (111) crystal plane of CuO, which may be caused by the oxidation of copper [11]. Accordingly, the XRD result demonstrated that the NiCu alloy nanoparticles were successfully formed on the surface of CNFs.

**Figure 4:**
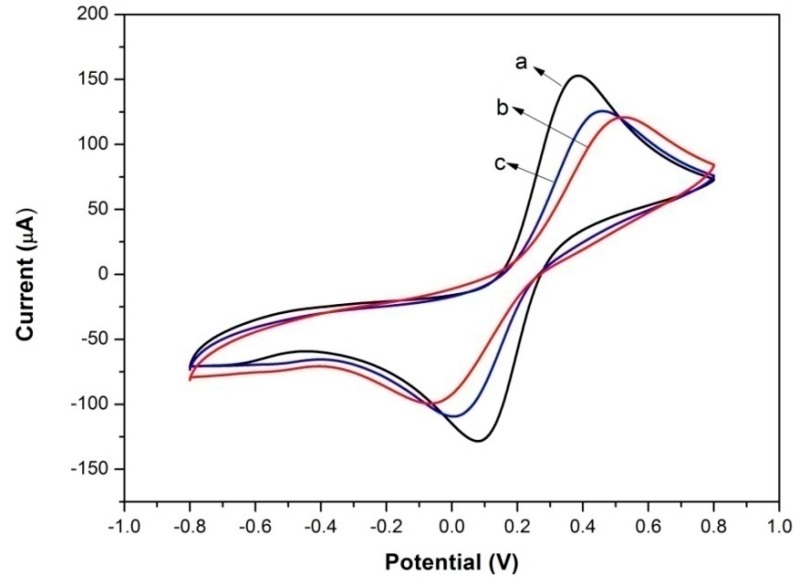
Cyclic voltammograms of (**a**) bare electrode; (**b**) Lac-Nafion/GCE; and (**c**) Lac-NiCuCNF-Nafion/GCE in 0.1 M KCl and 1 mM Fe(CN)_6_^3−/4−^ solution at 100 mV·s^−1^.

Cyclic voltammetry tests on bare electrode, Lac-Nafion/GCE and Lac-NiCuCNF-Nafion/GCE in 0.1 M KCl and 1 mM Fe(CN)_6_^3−/4−^ solution were carried out at 100 mV·s^−1^ to compare the interface resistances of different modified electrodes. It is seen from Figure 4 that all three modified electrodes displayed a pair of well-defined and stable redox peaks, in which the peak current value could reflect their interface resistances. Apparently, bare electrode (curve a) showed the highest current value, indicating the lowest interface resistance. For Lac-Nafion/GCE (curve b), the current value decreased significantly, and the interface resistance became larger than that of bare electrode because of the poor conductivity of Lac. Meanwhile, the peak-to-peak potential difference for Lac-Nafion/GCE also increased, which suggested that the electron transfer became more difficult as compared to that of bare electrode. Nevertheless, after the introduction of NiCuCNFs, the peak current value of the resultant Lac-NiCuCNF-Nafion/GCE (curve c) increased, and its peak-to-peak potential difference reduced. This demonstrated that the conductivity of Lac-NiCuCNF-Nafion composite was enhanced by adding NiCuCNFs.

### 3.2. Direct Electron Transfer of Lac-NiCuCNF-Nafion/GCE

**Figure 5:**
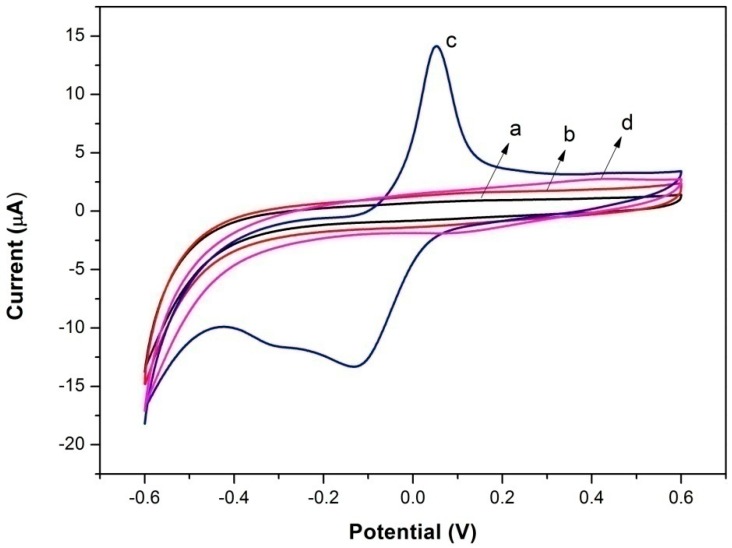
Cyclic voltammograms of (**a**) bare electrode; (**b**) Lac-Nafion/GE; (**c**) NiCuCNF-Nafion/GE; and (**d**) Lac-NiCuCNF-Nafion/GE in buffer solution at 100 mV·s^−1^.

Figure 5 shows the cyclic voltammograms of bare electrode, Lac-Nafion/GCE, NiCuCNF-Nafion/GCE and Lac-NiCuCNF-Nafion/GCE in blank acetate buffer solution. It is clear that the bare electrode (curve a) and Lac-Nafion/GCE (curve b) showed no redox peaks, indicating that there was no electron transfer occurred between the Lac and the GCE surface. NiCuCNF-Nafion/GCE showed a pair of redox peaks at 0.05 V and −0.13 V (curve c), which could be attributed to the redox reaction of NiCu bimetallic alloy. Notably, Lac-NiCuCNF-Nafion/GCE displayed a pair of redox peaks (curve d) located at *ca.* 0.4 V and 0.1 V (*vs.* Ag/AgCl), respectively, which could be ascribed to the direct electrochemistry behavior of Lac. The formal potential (E°′) is around 0.25 V and the peak-to-peak separation (ΔE_p_) is 300 mV at 100 mV·s^−1^. This demonstrated that the DET of Lac was achieved by the NiCuCNFs by linking Lac active center to GCE surface.

**Figure 6:**
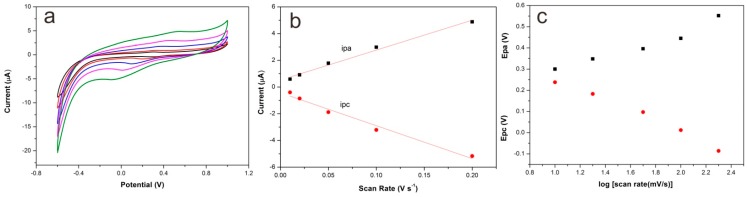
(**a**) Cyclic voltammograms of Lac-NiCuCNF-Nafion/GCE in acetate buffer solution at 10, 20, 50, 100 and 200 mV·s^−1^ (from inner to outer); (**b**) Calibration curve of the peak currents *vs.* scan rates; (**c**) Plot of anodic (Epa) and cathodic (Epc) peak potentials *vs.* log(scan rate).

Figure 6 depicts the effect of scan rate on the cyclic voltammograms of Lac-NiCuCNF-Nafion/GCE in pH 5.0 0.1 M acetate buffer solution. It is apparent that not only anodic peak currents but also cathodic peak currents became larger as the scan rate increases, as shown in Figure 6a. Besides, it can be seen from Figure 6b that the current values presented a linear increment with the ascent of scan rate, which suggested the entire electrochemical reaction existing on the surface of electrode was a surface-controlled electrochemical reaction process. It is noticeable that the anodic peak potentials (*E*pa) and cathodic peak potentials (*E*pc) shifted to more positive positions and more negative positions, respectively, with the change of scan rate, indicating the quasi-reversible electrochemical reaction process. Based on the Laviron equation [32], the charge-transfer coefficient (α) can be calculated by the following equation:

(1) $$\alpha =\frac{\delta_{\text{pa}}}{\delta_{\text{pa}}-\delta_{\text{pc}}}$$

where $\delta_{\text{pa}}$ and $\delta_{\text{pc}}$ are the anodic and cathodic slopes of linear plots of $E_{\text{pa}}$ and $E_{\text{pc}}$ *vs.* log(scan rate) (Figure 6c). Besides, slopes for the anodic and cathodic peaks can be calculated by the following equations:

(2) $$\delta_{\text{pa}}=\frac{2.3RT}{\left(1-\alpha \right)nF}$$

(3) $$\delta_{\text{pc}}=\frac{-2.3RT}{\alpha nF}$$

where *F* is the Faraday constant (96,500 C mol^−1^), *R* is the thermodynamic gas constant (8.314 J·K^−1^·mol^−1^), *T* is the temperature (298 K). From the above equations, α was calculated to be 0.5, *n* was 1. The electron transfer rate constant ($K_{\text{s}}$) of Lac at the Lac-NiCuCNF-Nafion/GCE can be calculated by the following equation:

(4) $$\text{log}(K_{\text{s}})=\alpha \log\left(1-\alpha \right)+\left(1-\alpha \right)\log\alpha -\log(RT/nF\nu )-\alpha \left(1-\alpha \right)(nF\Delta E\text{p}/2.3RT)$$

ΔEp was 430 mV at ν(scan rate) = 100 mV/s, *n* = 1, α *=* 0.5, the value of $K_{\text{s}}$ was estimated to be 1.23 s^−1^. The surface coverage (Г, in mol·cm^−2^) of electroactive species on the electrode was calculated by the formula *Q* = *nFA*Г, *Q* is the charge (C), *F* is the Faraday constant, *A* is the electrode area (cm^2^), and *n* is the transfer electron number. The surface coverage of electroactive laccase was calculated to be 2.38 × 10^−10^ mol·cm^−2^. This value is larger than the value reported in literature (1.3 × 10^−11^ mol·cm^−2^) [33], which suggests that more Lac was immobilized in the Lac-NiCuCNF-Nafion composite.

### 3.3. Electrocatalysis of Different Electrodes

**Figure 7:**
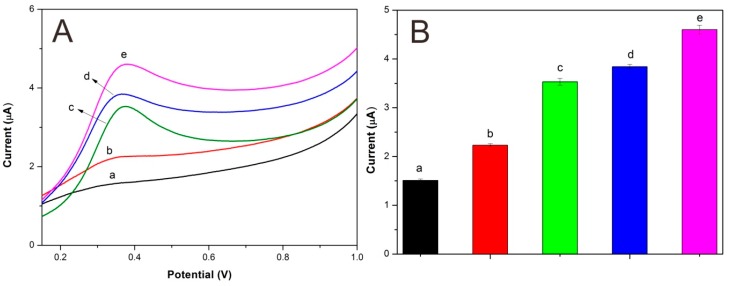
(**A**) Linear sweep voltammograms of different electrodes: (a) bare electrode, (b) Lac-Nafion/GCE, (c) Lac-CuCNF-Nafion/GCE, (d) Lac-NiCNF-Nafion/GCE, and (e) Lac-NiCuCNF-Nafion/GCE in acetate buffer solution with 1.59 µM ofhydroquinone at 50 mV·s^−1^; (**B**) Peak current value corresponding to every electrode.

Linear sweep voltammetry (LSV) was applied to study the biosensor response to hydroquinone. Figure 7 compares different modified electrodes in terms of their electrocatalysis toward 1.59 µM of hydroquinone. It is obvious that the bare electrode (curve a) showed a nearly steady curve, indicating its poor electrocatalysis toward hydroquinone. However, Lac-Nafion/GCE (curve b) showed an oxidation peak at around 0.35 V, which was caused by the catalysis of Lac towards hydroquinone. Lac-CuCNF-Nafion/GCE (curve c) and Lac-NiCNF-Nafion/GCE (curve d) both showed higher peak current values than Lac-Nafion/GCE, implying that the sensitivity of the electrode was improved by the addition of CuCNFs or NiCNFs. It is noticeable that among all modified electrodes, Lac-NiCuCNF-Nafion/GCE (curve e) showed the largest peak current value at around 0.38 V. This demonstrated that NiCuCNFs possessed stronger sensibilization than CuCNFs and NiCNFs, which could be explained by that NiCu bimetallic alloy had better electrochemical response than pure Ni or Cu during the electrochemical reaction process [11].

**Figure 8:**
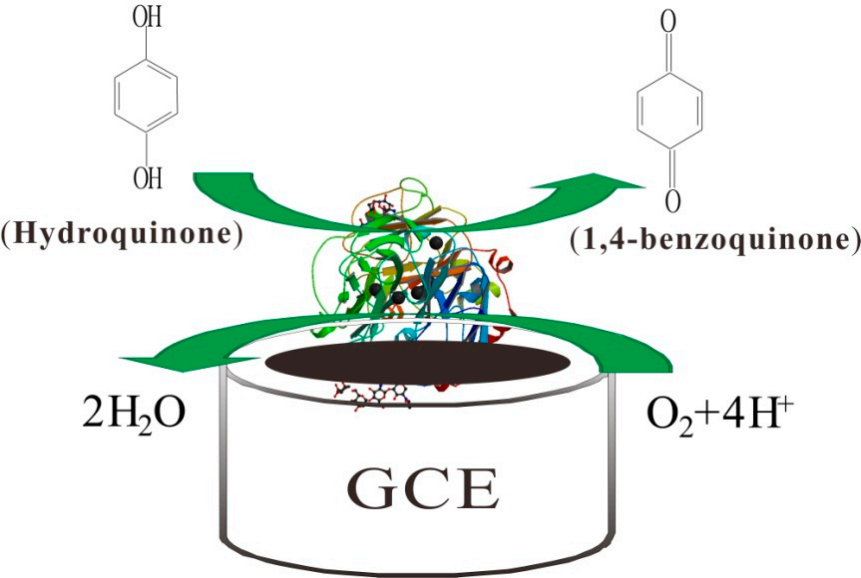
Schematic representation of the biosensing mechanism.

Figure 8 shows the possible reaction mechanism. Firstly, with the existence of molecular oxygen, the hydroquinone was oxidized to 1,4-benzoquinone by Lac. During this process, the Lac was reduced to its reduced state, which could be oxidized back to its oxidized state by oxygen. After that, the 1,4-benzoquinone, which obtained two electrons from the GCE, was electrochemically reduced to hydroquinone again on the GCE surface. It is a reversible cyclic reaction process.

### 3.4. Analytical Characteristics of Lac-NiCuCNF-Nafion/GCE

**Figure 9:**
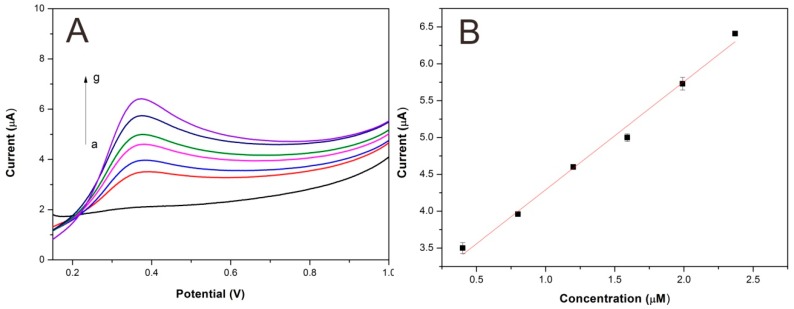
(**A**) Linear sweep voltammograms of Lac-NiCuCNF-Nafion/GCE for (a) blank buffer solution and hydroquinone solutions with different concentrations: (b) 0.40; (c) 0.80; (d) 1.20; (e) 1.59; (f) 1.99; (g) 2.37 µM at 50 mV·s^−1^; (**B**) calibration curve for hydroquinone.

Figure 9 shows the linear sweep voltammograms for hydroquinone using Lac-NiCuCNF-Nafion/ GCE. It can be seen that the peak current value increased simultaneously with the increase of hydroquinone concentration. According to the method reported in the literature [34], the Lac-NiCuCNF-Nafion/GCE sensitivity was calculated to be 1.5 µA·µM^−1^, the detection limit (S/N = 3) was 90 nM. The detection linear range was 4 × 10^−7^–2.37 × 10^−6^ M (Δ*I* = 2.8(±0.1) + 1.5(±0.065)*C*; *r*^2^ = 0.9902), here Δ*I* is the peak current (µA) and *C* is the concentration (µM). Table 1 compares Lac-NiCuCNF-Nafion/GCE with other biosensors. It is manifest that Lac-NiCuCNF-Nafion/GCE showed satisfactory detection results.

**Table 1:** Comparison of hydroquinone analytical performance of different sensors.

Electrode	Detection Limit (µM)	Linear Range (µM)	Reference
GCE/MWCNT/CoPc electrode	0.1600	0.99–8.30	[35]
Au-SAMmix-HRP electrode	1.2600	5.00–30.00	[36]
dsDNA/PANI/CTS/GCE	0.9600	1.25–320.00	[24]
GCE-PEI-AuNP-LAC	0.2100	2.90–22.00	[37]
Lac-NiCuCNF-Nafion/GCE	0.0900	0.40–2.37	this work

### 3.5. Repeatability, Reproducibility, Anti-Interference and Stability of Lac-NiCuCNF-Nafion/GCE

The repeatability, reproducibility, anti-interference and stability of Lac-NiCuCNF-Nafion/GCE was investigated by adding 1.5 µM hydroquinone in acetate buffer solution by LSV. The relative standard deviation (RSD) value for six successive measurements was 1.8%, implying a good repeatability. To study the reproducibility of biosensor, five Lac-NiCuCNF-Nafion/GCE modified electrodes were respectively prepared under the same condition and the RSD value was calculated to be 3.7%, which demonstrated that the Lac-NiCuCNF-Nafion/GCE possessed acceptable reproducibility.

**Figure 10:**
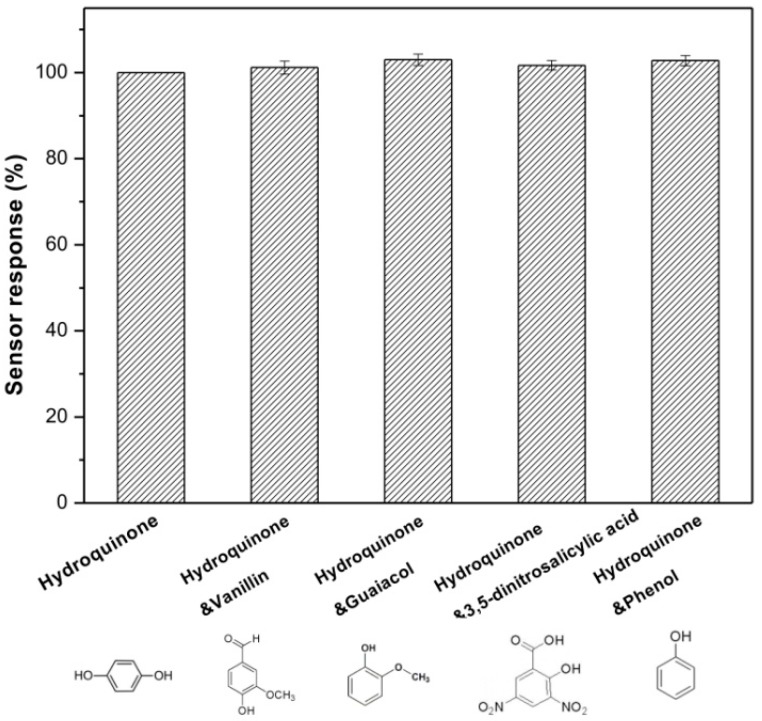
Anti-interference property of biosensor in buffer solution containing different interferents.

To investigate the anti-interference properties of Lac-NiCuCNF-Nafion/GCE, its LSV response towards different phenolic compounds were examined. It can be seen from Figure 10 that before adding 1.5 µM interferents, the current response of modified electrode to 1.5 µM hydroquinone was set as 100%. While, after each 1.5 µM interferent was added in the solution respectively, the current responses all increased slightly. However, the relative response values were negligible, which indicated that the Lac-NiCuCNF-Nafion/GCE possessed satisfactory anti-interference properties.

**Figure 11:**
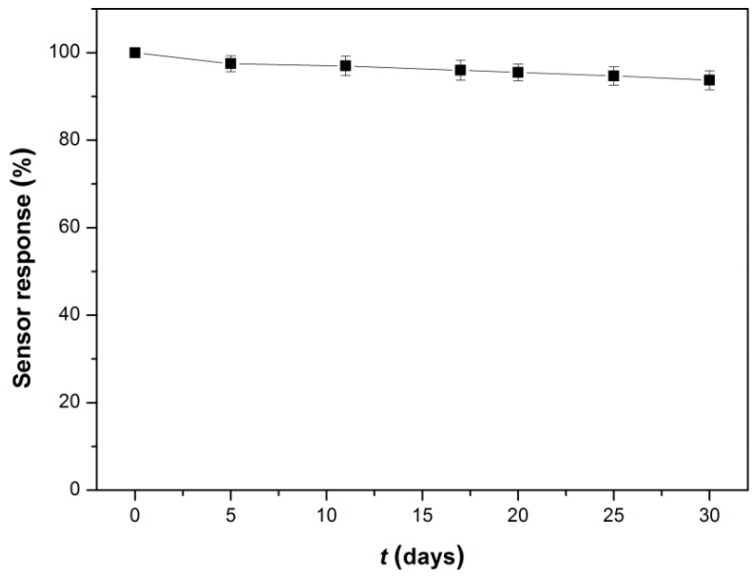
Storage stability of the biosensor.

The stability of Lac-NiCuCNF-Nafion/GCE biosensor was also studied, and the result is shown in Figure 11, it is clearly seen that through a long-term (30 days) storage in air at 4 °C, the current response value of Lac-NiCuCNF-Nafion/GCE still maintained 93.7% of the initial value, indicating the excellent stability of Lac-NiCuCNF-Nafion/GCE. These results demonstrated that the Lac-NiCuCNF-Nafion/GCE showed good repeatability, reproducibility, anti-interference and stability.

### 3.6. Real Water Sample Analysis

The practical application of the Lac-NiCuCNF-Nafion/GCE biosensor was also investigated by using untreated water from Lake Raleigh (Raleigh, NC, USA). Herein, a recovery experiment was used and repeated for five times and the results are displayed in Table 2. The recovery values for the five tests were 95.3%, 101.3%, 97.3%, 92.0% and 94.0%, respectively, and the RSD for these values was only 3.7%, demonstrating the successful application of the novel Lac-NiCuCNF-Nafion/GCE biosensor in real water sample analysis.

**Table 2:** Hydroquinone detection in lake water (*n* = 5).

Sample ^a^	C_added_ (µM)	C_found_ (µM)	Recovery (%)	RSD (%)
1	1.50	1.43	95.30	3.70
		1.52	101.30	
		1.46	97.30	
		1.38	92.00	
		1.41	94.00	

^a^ 1: Lake Raleigh water.

## 4. Conclusions

In summary, NiCuCNFs were synthesized by a combined method of electrospinning and thermal treatment. Subsequently, NiCuCNFs with Lac, Nafion and a GCE were integrated to construct a novel biosensing platform. The electrochemical experiments indicated that the DETof Lac could be achieved by NiCuCNFs. The resultant Lac-NiCuCNF-Nafion/GCE biosensor showed stronger electrocatalysis toward hydroquinone than Lac-CuCNF-Nafion/GCE and Lac-NiCNF-Nafion/GCE due to the better sensibilization of NiCuCNFs. The as-prepared biosensor was employed to detect hydroquinone and showed satisfactory detection results with a low detection limit, high sensitivity and wide linear range. Besides, the biosensor also showed good repeatability, reproducibility, anti-interference properties and stability. The biosensor was further employed to detect hydroquinone in lake water samples and achieved satisfactory recoveries. NiCuCNFs thus offer new opportunities for the construction of other enzyme-based biosensors for the analysis of environmental pollutants in the future.
